# miR-140-5p regulates the odontoblastic differentiation of dental pulp stem cells via the Wnt1/β-catenin signaling pathway

**DOI:** 10.1186/s13287-019-1344-4

**Published:** 2019-07-29

**Authors:** Xiaohui Lu, Xi Chen, Jing Xing, Min Lian, Dan Huang, Yuanzhou Lu, Guijuan Feng, Xingmei Feng

**Affiliations:** 1 Department of Stomatology, Affiliated Hospital of Nantong University, 20 Xisi Road, Nantong, 226001 Jiangsu China; 2 Department of Stomatology, Stomatological Hospital of Zhenjiang, Zhenjiang, Jiangsu China; 3 Department of Cardiology, The People’s Hospital of Tongzhou, Nantong, Jiangsu China

**Keywords:** miR-140-5p, Differentiation, Dental pulp stem cells, Wnt1, β-Catenin

## Abstract

**Background:**

MicroRNAs (miRNAs) play a key role in regulating cell differentiation. In the present study, we aimed to explore the role of miR-140-5p in odontoblastic differentiation of dental pulp stem cells (DPSCs).

**Methods:**

DPSCs from normal human impacted third molars were isolated and cultured. After overexpression or silencing of miR-140-5p in DPSCs, activity, proliferation, and odontoblastic differentiation of DPSCs were evaluated. The possible target gene of miR-140-5p was verified by luciferase reporter gene assay. Using gene transfection technology, RT-CPR, and Western blot to confirm miR-140-5p regulates the odontoblastic differentiation of DPSCs through Wnt1/β-catenin signaling.

**Results:**

We found the expression of miR-140-5p decreased in the differentiated DPSCs for odontoblastic cells, and at the same time, the expressions of Wnt1 and β-catenin increased. Wnt1 was the target gene of miR-140-5p which was confirmed by luciferase reporter gene system. miR-140-5p overexpression suppressed the expression of Wnt1. miR-140-5p inhibitor could promote the odontoblastic differentiation of DPSCs. miR-140-5p mimic could weaken the odontoblastic differentiation of DPSCs, which could be reversed by the overexpression of Wnt1.

**Conclusion:**

Our data demonstrated that miR-140-5p regulates the odontoblastic differentiation of DPSCs via targeting Wnt1/β-catenin signaling. Therefore, miR-140-5p might be a molecular target to regulate the odontoblastic differentiation for the therapeutic agents in dental medicine.

**Electronic supplementary material:**

The online version of this article (10.1186/s13287-019-1344-4) contains supplementary material, which is available to authorized users.

## Background

The exposure of the pulp can occur as a consequence of deep caries, traumatic injuries, or iatrogenic injury and often results in inflammation or death of the pulp [1]. Endodontically treated teeth become devitalized, brittle, and susceptible to postoperative fracture or other complications, including re-infections [2]. Therefore, new strategies are needed in order to try to regenerate the original tissue and subsequently restore the dental pulp properties [1]. Dental pulp stem cells (DPSCs), as a type of fibroblast, are present in the pulp. It has multidirectional differentiation potential and is responsible for the self-repairing function of the dental pulp tissue. DPSCs have become a hot spot in stomatological research in the world [3]. Studies have shown that DPSCs can differentiate into osteoblasts, odontoblasts, adipocytes, muscle cells, and nerve cells, with strong proliferative capacity and high differentiation potential [4]. The regulation mechanism of DPSC-directed differentiation has been a hot topic in the field, and there are still many difficulties to be solved. This also greatly limits the clinical application of DPSCs.

MicroRNAs are one of the most abundant gene-regulating molecules that may affect the export of many protein-coding genes in multicellular organisms. There is increasing evidence that microRNAs are involved in a wide range of underlying cellular processes such as cell differentiation, proliferation, growth, migration, and apoptosis as well as carcinogenesis or inhibition of cancers [5–8]. miR-140-5p is a member of the microRNAs family. Recent studies have shown that miR-140-5p is involved in the proliferation and differentiation of cells [9–12]. In the preliminary experiments of inducing differentiation of DPSCs into odontoblasts, we observed the expression of miR-140-5p was significantly decreased in the induced differentiation group. The target gene of miR-140-5p was analyzed and predicted using the online software targetscan (http://www.targetscan.org). The results showed that Wnt1 may be one of the target genes of miR-140-5p (more detail is shown in Additional file 1). Our previous study found β-catenin was also involved in the odontoblast differentiation of DPSCs [6]. The Wnt/β-catenin signaling pathway is widely distributed in invertebrates and vertebrates and is a highly conserved signaling pathway during species evolution [13]. Wnt/β-catenin signaling plays a crucial role in the early development of embryos, organ formation, tissue regeneration, and other physiological processes [14–19]. In summary, this study proposes that miR-140-5p regulates the odontoblastic differentiation of DPSCs via targeting Wnt1/β-catenin signaling. In this study, DPSCs from normal human impacted third molars were isolated and cultured. After overexpression or silencing of miR-140-5p in DPSCs, MTT assay and Ki67 immunofluorescence were used to evaluate the effect of miR-140-5p on the activity and proliferation of DPSCs. The possible target gene of miR-140-5p was verified by luciferase reporter gene assay. Using gene transfection technology, RT-CPR, and Western blot to confirm miR-140-5p regulates the odontoblastic differentiation of DPSCs through Wnt1/β-catenin signaling.

## Materials and methods

### Cell culture

The cell culture process was performed as described in our previous studies [6, 20] and approved by the Ethics Committee of the Affiliated Hospital of Nantong University. Briefly, normal human impacted third molars were collected from patients of 14~22 years of age (*n* = 10) after they had given informed consent. The pulp was digested in a solution of 3 mg/mL collagenase type I for 1 h at 37 °C. Single-cell suspensions of dental pulp were seeded into 25-cm^2^ culture dishes and cultured in Dulbecco’s modified Eagle medium (DMEM) supplemented with 10% fetal bovine serum (FBS), 100 U/mL penicillin, and 100 μg/mL streptomycin at 37 °C under 5% CO_2_. Cells were passaged at the ratio of 1:3 when they reached 85–90% confluence. The cell populations were characterized by positive staining with anti-CD34, STRO-1, and c-kit, and by the absence of CD45. Cells from the fourth passage were used in all experiments.

### Odontoblastic differentiation

The odontoblastic differentiation process was performed as described in our previous study [6]. Briefly, DPSCs from the fourth passages were cultured in odontogenic differentiation medium containing a Minimum Essential Medium (Invitrogen, Carlsbad, CA), 15% FBS, 10 mmol/L β-glycerophosphate, 50 mg/mL α-ascorbic acid, 10 nmol/L dexamethasone (Sigma-Aldrich, St. Louis, MO), 0.292 mg/mL glutamine, 100 μg/mL streptomycin, and 100 U/mL penicillin for 14 days.

### The expression of miR-140-5p was detected by qRT-PCR

The RNA in cells was extracted using TaqMan miRNA isolation kit, then the expression of mature miR-140-5p was detected by the TaqMan miRNA assay and TaqMan Universal PCR Master Mix (Applied Biosystems, Foster City, CA, USA). U6 was used as the internal reference. The qRT-PCR relative quantitative method was used to analyze the experimental result.

### Cell transfection

The miR-140-5p mimic (abm, Canada), miR-140-5p inhibitor (abm, Canada), corresponding negative control, restructuring plasmid pcDNA3.1-Wnt1, and empty vector control pcDNA3.1 (GenePharma, Shanghai, China) were transfected into DPSCs using Lipofectamine™ 2000 (Invitrogen, Carlsbad, CA, USA) according to the manufacturer’s instruction.

### Alizarin red S staining

The staining process was performed as described in our previous study [21]. The differentiated cells were fixed with 4% paraformaldehyde (PFA) for 30 min and washed with PBS. Subsequently, cells were incubated with 2% Alizarin red S solution for 10 min at room temperature under gentle agitation. Cells were then washed with deionized water to remove excess staining. Mineralization was quantified by extracting the Alizarin red S stain with 100 mM cetylpyridinium chloride solution (Sigma-Aldrich) at room temperature. The absorbance of the extracted Alizarin red S stain was measured at 570 nm.

### Cell activity assay

DPSC activity was measured by using methyl-thiazole-tetrazolium (MTT) assay as described by Peng et al. [22]. MTT (AMRESCO) solution (5 mg/mL) was added to the cultures (10 μL MTT solution per 100 μL medium), followed by the incubation with 5% CO_2_ at 37 °C for 4 h. A solution of sodium dodecyl sulfate (SDS) was added to the cultures, which was incubated for 20 h at 37 °C in 5% CO_2_. The optical density (OD) was read on a Universal Microplate Reader (Synergy 2, Bio-Tek Instruments, Inc., USA) using a test wavelength of 570 nm.

### Cell proliferation assay

DPSC proliferation was measured by using Ki67 immunofluorescence. Cells were first fixed in 4% PFA, followed by incubation in hydrogen peroxide, and then incubated with the rat anti-Ki67 antibody (1:800, Abcam, Cambridge, UK) overnight. The following day, the cells were labeled the secondary antibody with Alexa Fluor 568-conjugated goat anti-rat (1:1000, Molecular Probes). The cell nuclei were counter-stained with Hoechst33342 for 30 min. Positive cells were observed under a fluorescent microscope.

### Western blot analysis

The cell total protein analysis process was performed as described in our previous study [20]. Briefly, the separated proteins were transferred onto polyvinylidene difluoride membranes, then the membranes were blocked with 5% nonfat milk and incubated with primary antibodies and second antibodies in turn. The following primary antibodies were used: rabbit anti-Wnt1(1:1000, Abcam), rabbit anti-β-catenin (1:1000, Abcam), rabbit anti-dentin sialophosphoprotein (DSPP) (1:1000, Abcam), rabbit anti-dentin matrix protein-1 (DMP-1) (1:1000, Abcam), and mouse anti-β-actin (1:1000, Santa Cruz). The second antibodies were goat-anti-rabbit or goat-anti-mouse horseradish peroxidase-conjugated IgG (1:1500, Abcam).

A Nuclear Protein Extraction Kit (Active Motif, Carlsbad, CA, USA) was used to extract the nuclear fractions according to the manufacturer’s instructions. The PVDF membranes were probed with specific antibodies: rabbit anti-β-catenin (1:1000, Abcam) and mouse anti-Lamin B1 (1:2000, Abcam) overnight at 4 °C. Then, they are followed by incubation with the second antibodies: goat-anti-rabbit or goat-anti-mouse horseradish peroxidase-conjugated IgG (1:1500, Abcam).

### The target gene of miR-140-5p was detected by luciferase reporter gene system

Online software TargetScan (http://www.targetscan.org) was used to predict the target gene of miR-140-5p, and the results showed Wnt1 might be the target gene. The wild-type (WT) 3′-UTR of Wnt1 mRNA has a putative miR-140-5p-binding site. The mutant-type (MuT) 3′-UTR of Wnt1 was constructed. The WT 3′-UTR of Wnt1 and MuT 3′-UTR of Wnt1 were inserted into pMIRGLO vectors (Promega Corporation, Madison, WI, USA). The miR-140-5p mimic, miR-negative control, WT 3′-UTR of Wnt1 vector, and MuT 3′-UTR of Wnt1 vector were co-transfected into the HEK293 cells. Following cell transfection for 48 h, luciferase activity was detected using a dual-luciferase reporter assay system (E1910; Promega Corporation).

### Statistical analysis

All the data were expressed by mean ± SD in this study and analyzed using statistical software SPSS 21.0. The difference among the groups was estimated by one-way ANOVA and compared using the LSD method. *P* < 0.05 was considered statistically significant.

## Results

### The expression of miR-140-5p is significantly decreased in the induced differentiation group

**Fig. 1:**
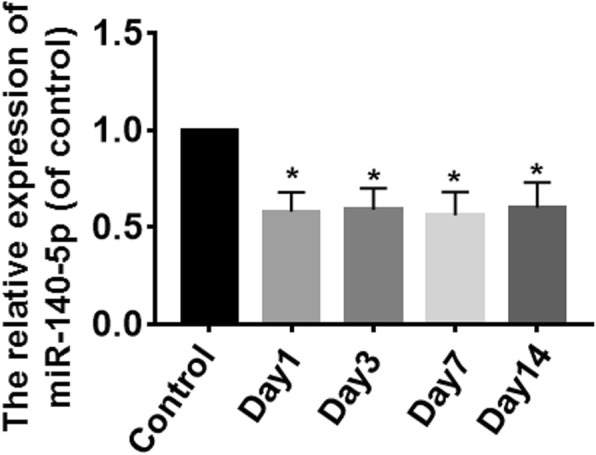
The expression of miR-140-5p was detected by qRT-PCR. After differentiation of day 1, day 3, day 7, and day 14, the expressions of miR-140-5p in the differentiation groups were lower than that in the control group. But there were no significant differences among day 1, day 3, day 7, and day 14. *vs. the control group, *P* < 0.05. *n* = 6

After differentiation of day 1, day 3, day 7, and day 14, the expressions of miR-140-5p in the differentiation groups were lower than that in the control group, and the difference was statistically significant (*P* < 0.05). But there were no significant differences among day 1, day 3, day 7, and day 14 (*P* > 0.05) (Fig. 1).

### miR-140-5p has no effects on cell activity and proliferation

**Fig. 2:**
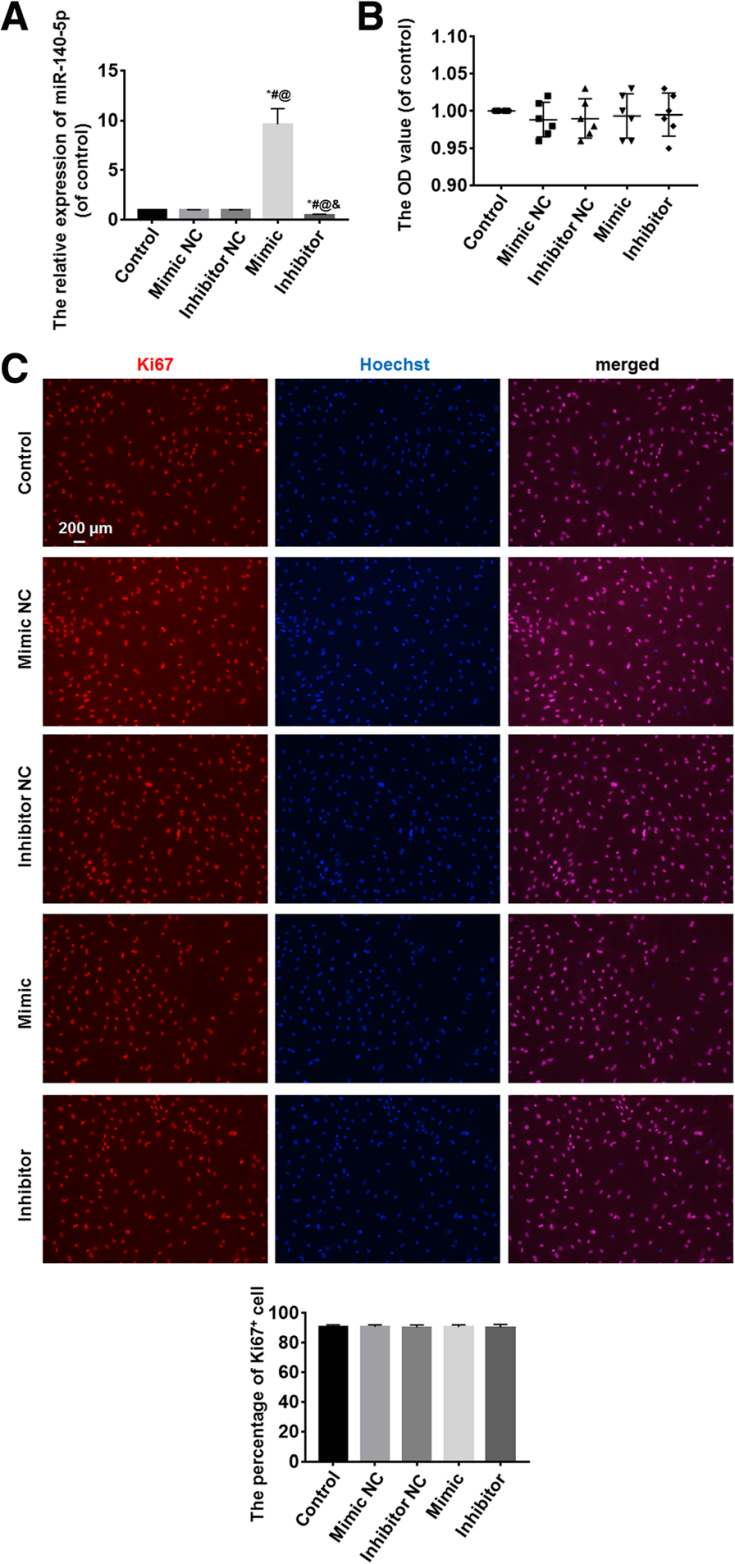
The effects of miR-140-5p on cell activity and proliferation. **a** The expression of miR-140-5p was detected by qRT-PCR. Compared with the control, mimic NC, and inhibitor NC groups, the expression of miR-140-5p increased in the mimic group and decreased in the inhibitor group. **b** The cell activity was measured by MTT. OD values of the control, mimic, mimic NC, inhibitor, and inhibitor NC groups had no differences. **c** The cell proliferation was measured by Ki67 immunofluorescence. There were no significant differences in Ki67^+^ cell number of the control, mimic, mimic NC, inhibitor, and inhibitor NC groups. *vs. the control group, *P* < 0.05; ^#^vs. the mimic NC group, *P* < 0.05; ^@^vs. the inhibitor NC group, *P* < 0.05; ^&^vs. the mimic group, *P* < 0.05. *n* = 6

Following cell transfection for 48 h, the expression of miR-140-5p was detected by qRT-PCR; the cell activity and *proliferation* were measured by MMT and Ki67 immunofluorescence, respectively. Compared with the control, mimic negative control (NC), and inhibitor NC groups, the expressions of miR-140-5p were increased in the mimic group and decreased in the inhibitor group, and the differences were statistically significant (*P* < 0.05) (Fig. 2a). There were no significant differences in OD values of the control, mimic NC, inhibitor NC, mimic, and inhibitor groups (*P* > 0.05) (Fig. 2b). There were also no significant differences in Ki67^+^ cell number in the five groups (*P* > 0.05) (Fig. 2c).

### miR-140-5p inhibit the odontoblastic differentiation of DPSCs

**Fig. 3:**
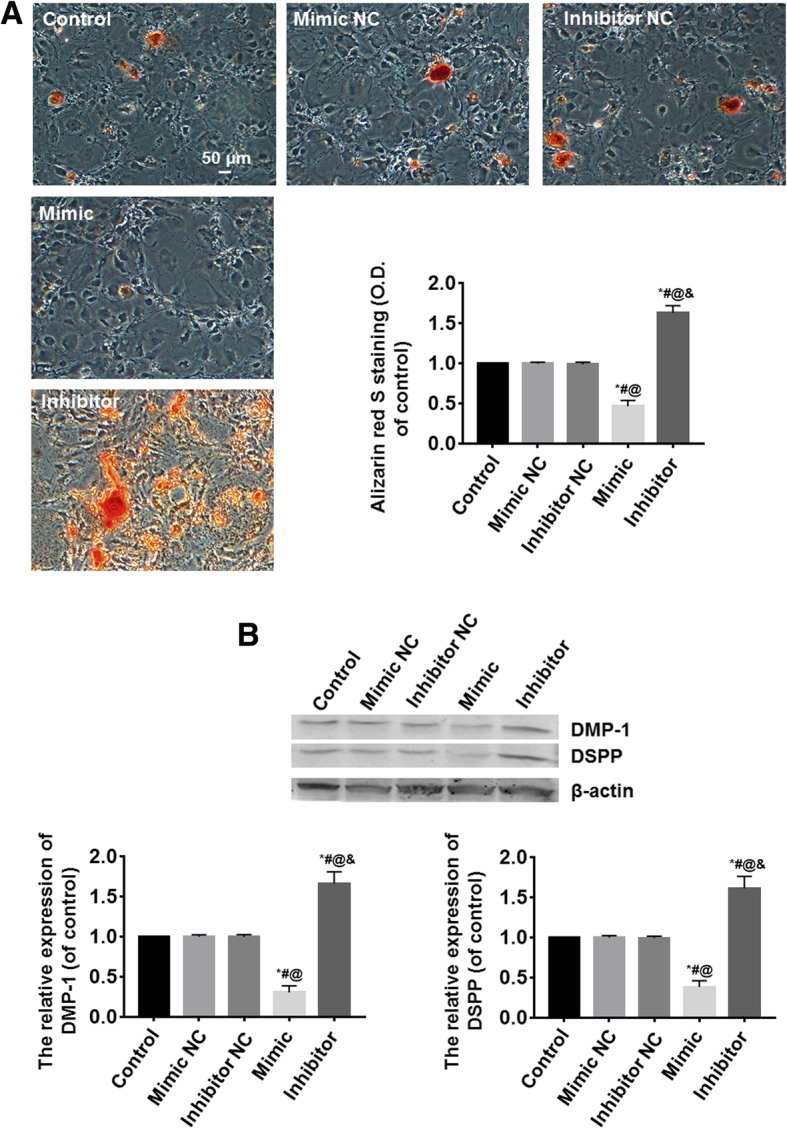
miR-140-5p inhibited the odontoblastic differentiation of DPSCs. **a** Alizarin red S staining showed the mineralized matrix deposition was the most in the inhibitor group and the least in the mimic group; the differences among the control, mimic NC, and inhibitor NC groups were not statistically significant. **b** The DMP-1 and DSPP proteins were detected by Western blot. The results showed the expressions of DSPP and DMP-1 proteins were the highest in the inhibitor group and the lowest in the mimic group; the differences among the control, mimic NC, and inhibitor NC groups were not statistically significant. *vs. the control group, *P* < 0.05; ^#^vs. the mimic NC group, *P* < 0.05; ^@^vs. the inhibitor NC group, *P* < 0.05; ^&^vs. the mimic group, *P* < 0.05. Bar = 50 μm. *n* = 6

Following cell transfection for 48 h, the cells were induced to odontoblastic differentiation for 14 days. Alizarin red S staining showed the mineralized matrix deposition was the most in the inhibitor group and the least in the mimic group (*P* < 0.05); the differences among the control, mimic NC, and inhibitor NC groups were not statistically significant (*P* > 0.05) (Fig. 3a). DSPP and DMP-1 proteins, marker of odontoblast, were detected by Western blot (see Additional file 2). The results showed the expressions of DSPP and DMP-1 proteins were the highest in the inhibitor group and the lowest in the mimic group (*P* < 0.05); the differences among the control, mimic NC, and inhibitor NC groups were not statistically significant (*P* > 0.05) (Fig. 3b).

### Wnt1 was the target gene of miR-140-5p

**Fig. 4:**
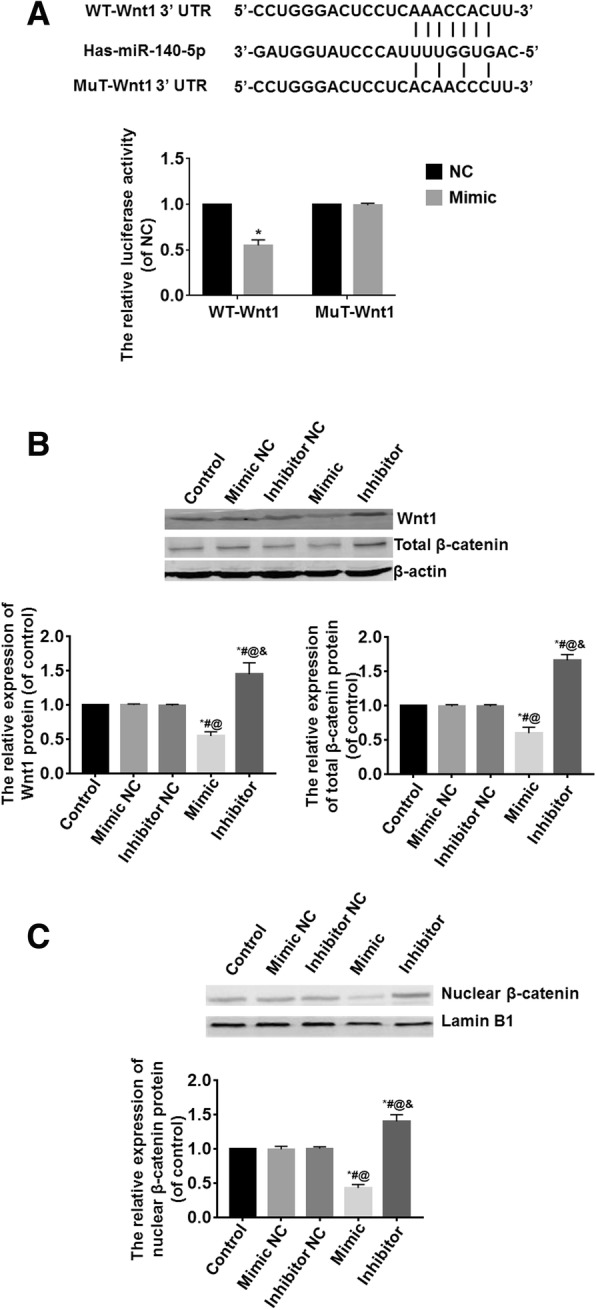
Wnt1 was the target gene of miR-140-5p. **a** Luciferase reporter gene detection results suggested that the luciferase activity of the mimic group was significantly lower than that of the mimic NC group. **b** Western blot analysis showed the expressions of Wnt1 and total β-catenin proteins were the highest in the inhibitor group and the lowest in the mimic group; the differences among the control, mimic NC, and inhibitor NC groups were not statistically significant. **c** Western blot analysis showed the expression of nuclear β-catenin protein was the highest in the inhibitor group and the lowest in the mimic group; the differences among the control, mimic NC, and inhibitor NC groups were not statistically significant. *vs. the control group, *P* < 0.05; ^#^vs. the mimic NC group, *P* < 0.05; ^@^vs. the inhibitor NC group, *P* < 0.05; ^&^vs. the mimic group, *P* < 0.05. *n* = 6

Luciferase reporter gene detection results suggested that the luciferase activity of the mimic group was significantly lower than that of the mimic NC group (*P* < 0.05) (Fig. 4a). Western blot analysis showed the expressions of Wnt1 and total or nuclear β-catenin proteins were the highest in the inhibitor group and the lowest in the mimic group (*P* < 0.05); the differences among the control, mimic NC, and inhibitor NC groups were not statistically significant (*P* > 0.05) (Fig. 4b, c).

### Overexpression of Wnt1 reverses the inhibition of odontoblastic differentiation induced by miR-140-5p

**Fig. 5:**
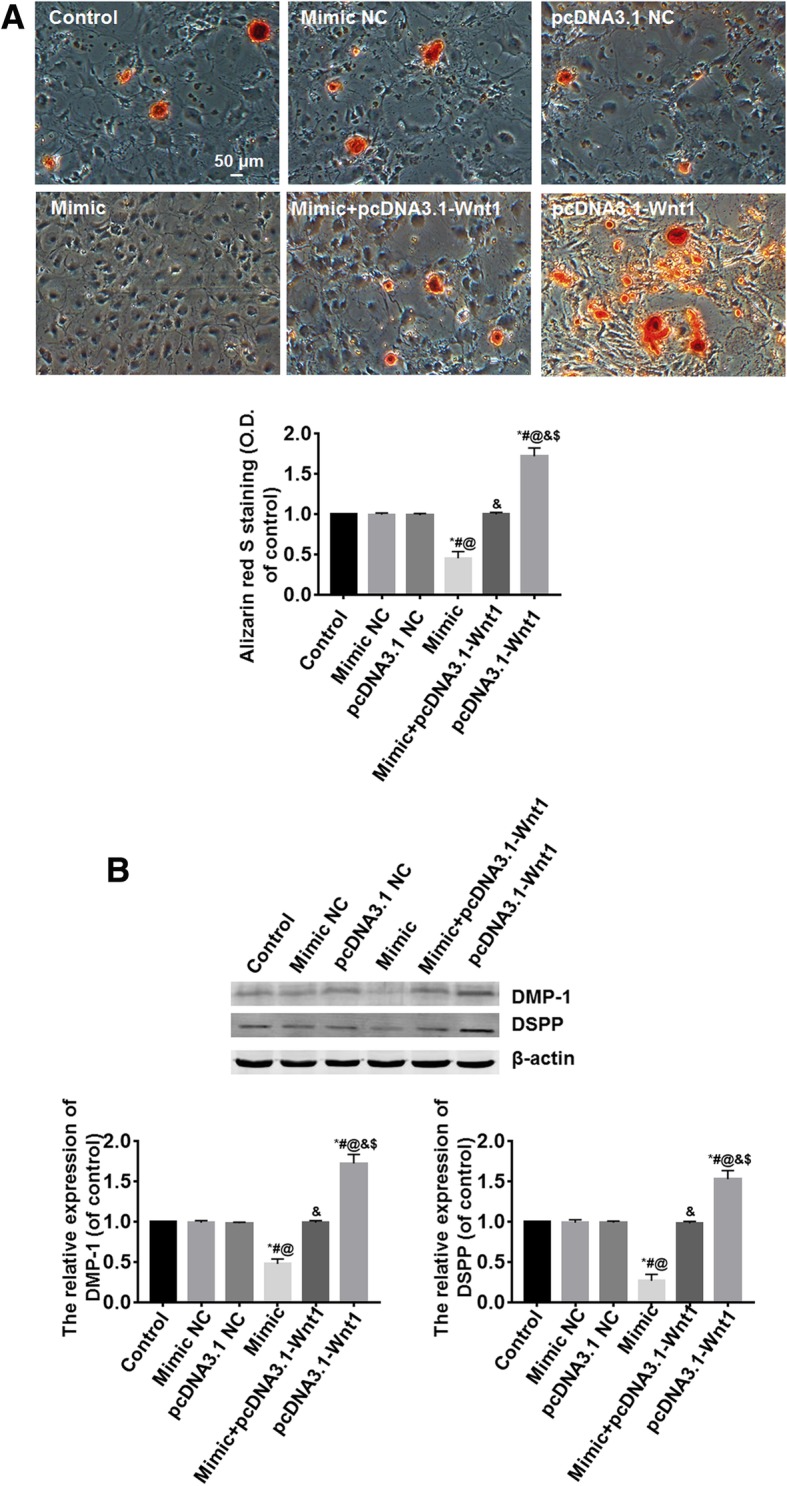
Overexpression of Wnt1 reversed the inhibition of odontoblastic differentiation induced by miR-140-5p. **a** Alizarin red S staining showed the mineralized matrix deposition was the least in the mimic group and the most in the pcDNA3.1-Wnt1 group. The percent of positive cells in the pcDNA3.1-Wnt1+mimic group was similar to that of the control, mimic NC, and pcDNA3.1 NC groups; the differences among them were not statistically significant. **b** Western blot analysis showed the expressions of DSPP and DMP-1 proteins were the highest in the pcDNA3.1-Wnt1 group and the lowest in the mimic group. The expressions of DSPP and DMP-1 proteins in the pcDNA3.1-Wnt1+mimic group were similar to those of the control, mimic NC, and pcDNA3.1 NC groups; the differences among them were not statistically significant. *vs. the control group, *P* < 0.05; ^#^vs. the mimic NC group, *P* < 0.05; ^@^vs. the pcDNA3.1 NC group, *P* < 0.05; ^&^vs. the mimic group, *P* < 0.05; ^$^vs. the mimic+pcDNA3.1-Wnt1 group, *P* < 0.05. *n* = 6

Following cell transfection for 48 h, the cells were induced to odontoblastic differentiation for 14 days. Alizarin red S staining showed the mineralized matrix deposition was the least in the mimic group and the most in the pcDNA3.1-Wnt1 group (*P* < 0.05). The number of positive cells in the mimic+pcDNA3.1-Wnt1 group was similar to that of the control, mimic NC, and pcDNA3.1 NC groups; the differences among them were not statistically significant (*P* > 0.05) (Fig. 5a). DSPP and DMP-1 are necessary for the proper biomineralization of cementum, dentin, or enamel, which are usually used as the markers of odontoblastic differentiation. Western blot analysis showed the expressions of DMP-1 and DSPP proteins were the highest in the pcDNA3.1-Wnt1 group and the lowest in the mimic group (*P* < 0.05). The expressions of DMP-1 and DSPP proteins in the mimic+pcDNA3.1-Wnt1 group were similar to those of the control, mimic NC, and pcDNA3.1 NC groups; the differences among them were not statistically significant (*P* > 0.05) (Fig. 5b).

## Discussion

In this study, first, we found the expression of miR-140-5p was significantly decreased in the induced odontoblast differentiation. Based on this result, we downregulated or overexpressed miR-140-5p in DPSCs and found miR-140-5p knockdown upregulated odontoblast differentiation, while miR-140-5p overexpression had a negative effect on odontoblast differentiation in DPSCs. Furthermore, Wnt1, the target gene of miR-140-5p, was confirmed. We approved that miR-140-5p regulated the odontoblast differentiation of DPSCs through the Wnt/β-catenin pathway.

DPSCs can be simply isolated from freshly extracted teeth. These cells can be highly proliferated and multilineage-differentiated. Moreover, their clinical utility is attributable to their simplicity and convenience of isolation, lack of ethical controversy, and low immunogenicity and is used as autologous transplants for dentin regeneration and periodontal tissue regeneration [20]. However, how to regulate the directed differentiation of DPSCs is still a major problem for researchers. miR-140-5p is a member of the microRNAs family. Recent studies have shown that miR-140-5p is involved in the proliferation and differentiation of cells [9–12]. In the study of osteogenic or adipogenic differentiation of human adipose-derived mesenchymal stem cells, it was found that low expression of miR-140-5p could promote osteogenic differentiation [23]. Hwang et al. reported that miR-140-5p suppressed BMP2-mediated osteogenesis in undifferentiated human mesenchymal stem cells [11]. Sun et al. found miR-140-5p-mediated regulation of the proliferation and differentiation in DPSCs occurred through the lipopolysaccharide/Toll-like receptor 4 signaling pathway. Their results showed overexpression of miR-140-5p enhanced proliferation of DPSCs and inhibited DPSC differentiation, whereas suppression of miR-140-5p produced the opposite effects [12]. Our results indicated that low expression of miR-140-5p could promote odontoblast differentiation of DPSCs, whereas overexpression of miR-140-5p inhibited odontoblast differentiation, but the expression of miR-140-5p had no effect on the activity and proliferation of DPSCs, which was different from Sun et al. [12]; this difference may be due to the different detection condition. All the results indicated that miR-140-5p was a key miRNA that regulated DPSC proliferation and differentiation.

Furthermore, Wnt1, the target gene of miR-140-5p was confirmed in our study by luciferase reporter gene detection system. Western blot analysis showed the overexpression of miR-140-5p inhibited the expressions of Wnt1 and β-catenin, whereas the suppression of miR-140-5p produced the opposite effects. The overexpression of Wnt1 could reverse the inhibition of odontoblastic differentiation induced by miR-140-5p. The classical Wnt/β-catenin signaling pathway plays a crucial role in the early development of embryos, organ formation, tissue regeneration, and other physiological processes [14–19]. Meanwhile, more and more researches support that the Wnt/β-catenin signaling pathway regulates stem cell self-renewal, proliferation, and differentiation [24–26] and was involved in dental tissue development [27–29]. Our previous study found β-catenin was involved in the odontoblast differentiation of DPSCs, too [6]. In this study, the total and nuclear β-catenin protein levels were the highest in the inhibitor group and the lowest in the mimic group, which indicated miR-140-5p was involved in β-catenin activation. Combining the results of this study, it was believed that miR-140-5p regulated the odontoblast differentiation of DPSCs via the Wnt1/β-catenin signaling pathway. Since the expression level of miR-140-5p is naturally downregulated during odontoblastic differentiation of DPSCs, whether it is still meaningful to deliver inhibitor to the site of pulp regeneration needs further studies to confirm it although the result in this study indicates that miR-140-5p inhibitor promotes the odontoblastic differentiation of DPSCs in vitro.

## Conclusion

In summary, our data demonstrated that miRNA-140-5p regulates the odontoblastic differentiation of DPSCs via targeting the Wnt1/β-catenin signaling pathway. Therefore, miRNA-140-5p might be a molecular target to regulate the odontoblastic differentiation for therapeutic agents in dental medicine.

## Additional files

Additional file 1: The outcome of target gene prediction for miR-140-5p. The results showed that Wnt1 may be one of the target genes of miR-140-5p (red part). (XLSX 58 kb)

Additional file 2: The original band of DMP-1 and DSPP in Figs. 3 and 5. The DMP-1 and DSPP proteins were detected by Western blot. (PPTX 308 kb)

## Data Availability

The datasets used and/or analyzed during the current study are available from the corresponding author on reasonable request.
