# Supplementary material for: miR-140-5p regulates the odontoblastic differentiation of dental pulp stem cells via the Wnt1/β-catenin signaling pathway
Source: Stem Cell Res Ther. 2019 Jul 29;10:226. doi: 10.1186/s13287-019-1344-4 (PMC6664499; doi:10.1186/s13287-019-1344-4)
Supplement: Supplementary file 2 — The original band of DMP-1 and DSPP in Figs. 3 and 5. The DMP-1 and DSPP proteins were detected by Western blot. (PPTX 308 kb) [file 13287_2019_1344_MOESM2_ESM.pptx]

## Slide 1
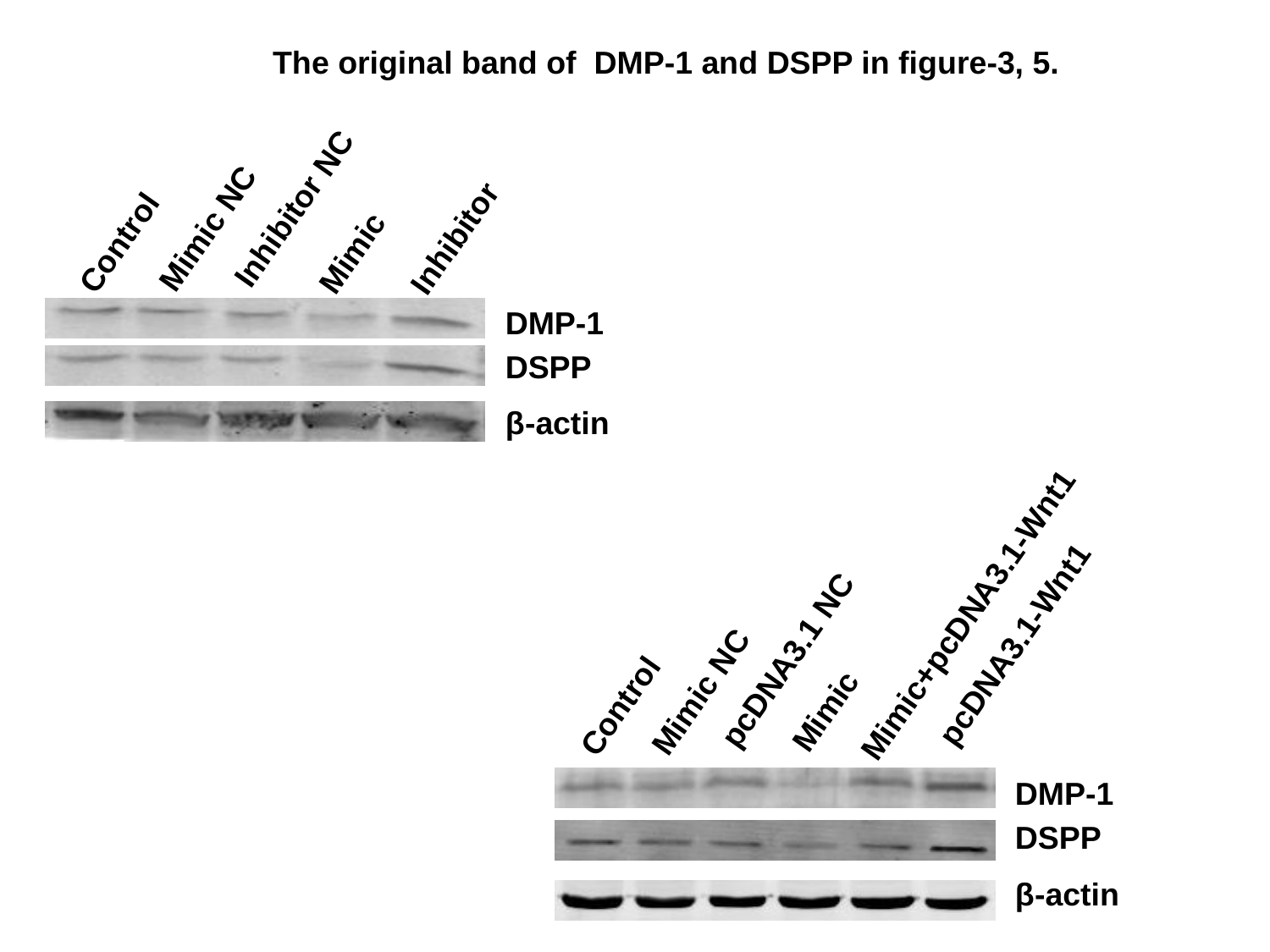

The original band of DMP-1 and DSPP in figure-3, 5.
Inhibitor NC
Mimic NC
Inhibitor
Control
Mimic
DMP-1
DSPP
β-actin
Mimic+pcDNA3.1-Wnt1
pcDNA3.1-Wnt1
pcDNA3.1 NC
Mimic NC
Control
Mimic
DMP-1
DSPP
β-actin
